# Computer Aided Quantification of Pathological Features for Flexor Tendon Pulleys on Microscopic Images

**DOI:** 10.1155/2013/914124

**Published:** 2013-06-06

**Authors:** Yung-Chun Liu, Hsin-Chen Chen, Hui-Hsuan Shih, Tai-Hua Yang, Hsiao-Bai Yang, Dee-Shan Yang, Fong-Chin Su, Yung-Nien Sun

**Affiliations:** ^1^Department of Computer Science & Information Engineering, National Cheng Kung University, Tainan 701, Taiwan; ^2^Medical Device Innovation Center, National Cheng Kung University, Tainan 701, Taiwan; ^3^Department of Neurosurgery, University of Pittsburgh, Pittsburgh, PA 15213, USA; ^4^Department of Biomedical Engineering, National Cheng Kung University, Tainan 701, Taiwan; ^5^Orthopedic Biomechanics Laboratory, Division of Orthopedic Research, Mayo Clinic Rochester, Rochester, MN 55905, USA; ^6^Department of Pathology, Medical College, National Cheng Kung University, Tainan 701, Taiwan; ^7^Department of Pathology, Ton-Yen General Hospital, Hsinchu 302, Taiwan; ^8^Department of Orthopedic Surgery, Ton-Yen General Hospital, Hsinchu 302, Taiwan

## Abstract

Quantifying the pathological features of flexor tendon pulleys is essential for grading the trigger finger since it provides clinicians with objective evidence derived from microscopic images. Although manual grading is time consuming and dependent on the observer experience, there is a lack of image processing methods for automatically extracting pulley pathological features. In this paper, we design and develop a color-based image segmentation system to extract the color and shape features from pulley microscopic images. Two parameters which are the size ratio of abnormal tissue regions and the number ratio of abnormal nuclei are estimated as the pathological progression indices. The automatic quantification results show clear discrimination among different levels of diseased pulley specimens which are prone to misjudgments for human visual inspection. The proposed system provides a reliable and automatic way to obtain pathological parameters instead of manual evaluation which is with intra- and interoperator variability. Experiments with 290 microscopic images from 29 pulley specimens show good correspondence with pathologist expectations. Hence, the proposed system has great potential for assisting clinical experts in routine histopathological examinations.

## 1. Introduction

Trigger finger is a common medical condition which occurs when the sheath of finger flexor tendon thickens, causing unsmooth glide of the tendon. The affected finger usually yields pains, intermittent snapping (triggering), or actual locking (during flexion or extension) resulting in patient difficulty [1]. Although more than one potential cause has been described, the etiology of the trigger finger remains idiopathic [2]. In order to understand the real causes and risk factors of trigger finger, microscopic evaluation for various degrees of pathological change hence becomes a critical issue. 

The pathological mechanism in the flexor sheath has been reported as the fibrocartilaginous metaplasia (or chondroid metaplasia) of its “A1” pulley based on the histopathological analysis [3]. In a normal pulley, there is a dense, regular, and connective tissue that is composed of collagenous fibers in compact and parallel bundles. Generally, histopathological specimens of collagenous fibers appear eosinophilic and pink in color under hematoxylin and eosin (H&E) stain. Moreover, it can be observed from microscopic images that the fibroblasts of a normal pulley possess long rod-like nuclei between the longitudinal bundles. On the other hand, the pulley of a trigger finger usually demonstrates the phenomenon of fibrocartilaginous metaplasia (or chondroid metaplasia), which is characterized by the presence of chondrocytes (cartilage cells). The affected fibers thus contain round nuclei and sulfate proteoglycans appearing in blue/purple color under the H&E stain [4].

A good interpretation of microscopic image depends on the level of abnormality observed from a combination of good visual evaluation and theoretical knowledge by pathologists [5]. Such qualitative evaluation of pathological changes remains the most common approach to grade trigger finger. However, due to intra- and interobserver variability, the accuracy of the grading results is decreased and the reproducibility of the experiment is difficult to ensure. Moreover, some quantities, such as the amount of nuclei, are impractical to obtain by visually examining the entire microslide. These limitations increase the probability of making an inappropriate decision for follow-up therapy. The aim of this paper is to define two parameters that reflect the above-mentioned color and shape features of pulley specimens and develop an image analysis system for automatic and objective microscopic evaluation of the pulley pathological changes. 

Microscopic image analysis methods have been actively investigated because they provide the most direct information for evaluating morphological or functional changes of tissues of interest at the microscopic level. Tabesh et al. [6] proposed an automatic prostate cancer classification system to analyze the microscopy of the prostate cancer tissues with color features in the R, G, and B channels of the acquired images. However, as the acquired images are nonuniformly illuminated, their simple thresholding method is not directly applicable in our case. Wu et al. [7] proposed a live cell image segmentation method to directly segment the cell regions using gray level. However, in our case, the pink areas which represent the normal tissues and the purple areas which represent the diseased tissues show very close gray level in the acquired images. Only using the gray level information in separating the abnormal from normal tissues on the pulley microscopic image would likely give erroneous results. The Canny edge detector is also a popular way to detect the border of cells [8]. However, in our case, the Canny operator detects not only the borders of nuclei but also the borders of dark blue and noise areas. As we are only interested in the borders of nuclei, too many irrelevant edges detected by the Canny operator tend to make the postprocessing process tedious and increase the likelihood of detection errors.

**Figure 1:**
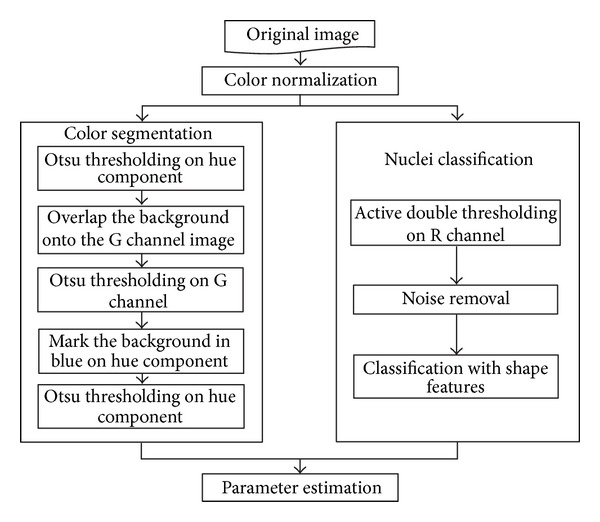
Flowchart of the proposed system.

In this paper, we propose an image analysis system to automatically quantify the pathological features of pulleys with trigger finger on microscopic images. Two parameters, which are the size ratio of abnormal tissue regions (parameter 1) and the number ratio of abnormal nuclei (parameter 2), are designed to reflect the severity of diseased tissues based on the pathologist suggestions.  Figure 1 shows the flowchart of the proposed method. First, the proposed system applies a color normalization to efficiently reduce the influence of nonuniform color distribution among the captured images. Then, the system adopts a three-stepped color segmentation process to extract normal and abnormal tissue regions from the hue-saturation-intensity (HSI) color space of the color-normalized image in order to calculate parameter 1. In addition, we design an active double thresholding algorithm to segment the nuclei and utilize a rule-based classifier based on nuclei shape properties to identify normal and abnormal nuclei for calculating parameter 2. Experiments demonstrate high correspondence between the automatically estimated parameters and the qualitative judgments of a pathologist.

## 2. Materials

The microscopic images of specimens in this study were provided by the laboratories in National Cheng Kung University Hospital and in Ton Yen General Hospital. The pathological pulley tissue specimens were obtained from the patients who were clinically diagnosed with trigger finger disease by orthopedists D. S. Yang and T. H. Yang. For pathological examination, all of the specimens followed the procedures of fixation in formalin, procession in graded alcohols and xylene, embedding in paraffin, cutting of sections with a microtome, and being stained with hematoxyline-eosin (H&E). The microtome was preset for a 5 *μ*m in thickness.

In these specimens, the normal pulley showed a dense regular fibrotic tissue. The collagenous fibers were arranged in compact, parallel bundles. Between the bundles were rows of modified fibroblasts with elongated spindle-shaped nuclei. The pathologic pulley tissue presented fibrocartilage metaplasia. It was composed of irregular connective tissue with fibrocartilaginous metaplasia (or chondroid metaplasia). In the H&E stained slides, the nuclei were dark blue in color and the collagenous fibers were pink in color. The fibrocartilaginous metaplastic (or chondroid metaplastic) tissue demonstrated more chondromyxoid materials (including hyaluronic acid, chondroitin sulfate, and proteoglycan) and showed blue or purple colors. Furthermore, nuclei of cartilage-like cells were round in shape. The prepared slides were first observed and graded according to the severity of myxoid metaplasia by pathologist H. B. Yang under a light microscope (Olympus, BX50). These specimens were also analyzed by the proposed system based on the above-mentioned color and shape features. The automatic evaluation results were then compared with the manually graded results.

## 3. Methods

### 3.1. Color Normalization

**Figure 2:**
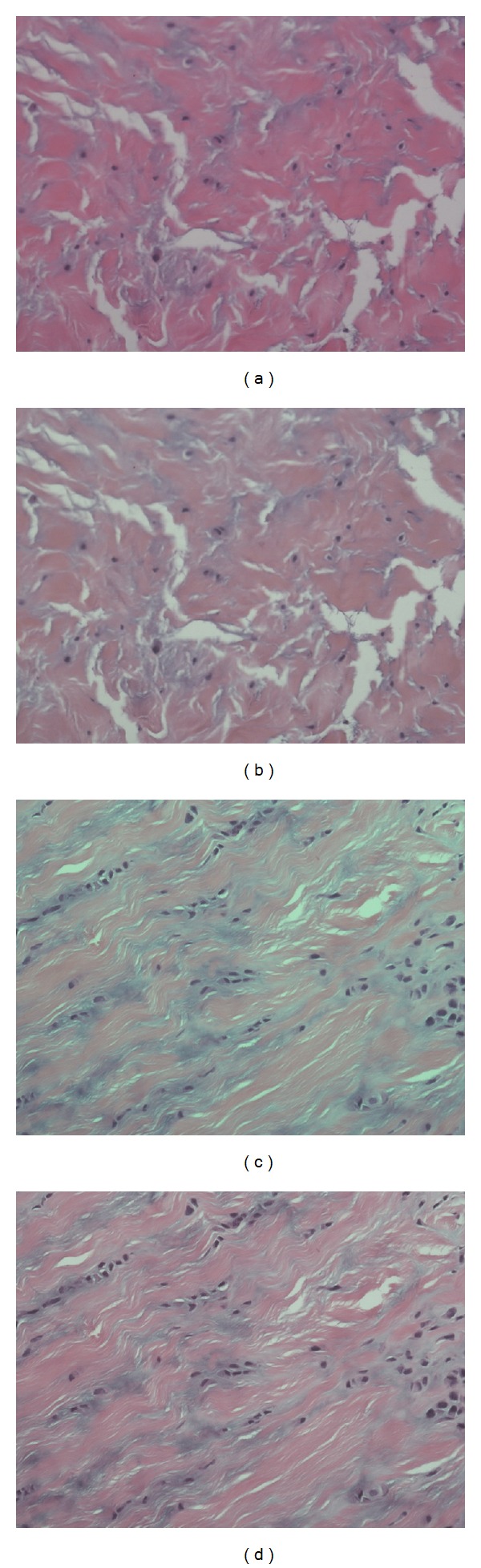
Color normalization. (a) and (c) are two original images from different specimens; (b) and (d) are results of (a) and (c) after performing color normalization, respectively.

The color normalization method is used to resolve the problem of nonuniform distribution in color and illumination of the acquired images, which are caused by the different staining and imaging conditions of the microscopic slices. As shown in Figures 2(a) and 2(c), color distributions of the two acquired images are quite different from each other. Color normalization will help to map these different microscopic images to a common image type with similar color distribution.

The color normalization method provided by Reinhard et al. [9] is adopted in this study. Initially, we must choose some standard images (target images) from the source image dataset with the following characteristics: the contrast ratio is high and the color of nuclei is dark blue. In other words, these standard images can show high contrast and can be used to categorize the various tissue types. We then normalize the input (or source) image to the color distribution of target images.

We transform the images from *RGB* color space into *LMS* color space by the following equation:

(1) $$\begin{array}{c} \left[\begin{array}{c} L\\ M\\ S \end{array}\right]=\left[\begin{array}{ccc} 0.3811 & 0.5783 & 0.0402\\ 0.1967 & 0.7244 & 0.0782\\ 0.0241 & 0.1288 & 0.8444 \end{array}\right]\left[\begin{array}{c} R\\ G\\ B \end{array}\right]. \end{array}$$

Because the data in this color space are often quite skewed, Reinhard et al. reduced skew error by converting the data to a logarithmic space by using (2):

(2) $$\begin{array}{c} L^{\prime }=\log L,\;\;M^{\prime }=\log M,\;\;\;S^{\prime }=\log S. \end{array}$$

Moreover, Ruderman et al. [10] suggested a transformation from *L*′*M*′*S*′ to *lαβ* through (3). It is because *lαβ* are the three orthogonal axes decomposed from *L*′*M*′*S*′ by using principle component analysis into the three most maximal directions (*lαβ*) decorrelating the *L*′*M*′*S*′ axes. In the experiments, the resulting color distribution of different tissues is more widely separated in *lαβ* color space than in the original *RGB* color space:

(3) $$\begin{array}{c} \left[\begin{array}{c} l\\ \alpha \\ \beta \end{array}\right]=\left[\begin{array}{ccc} \frac{1}{\sqrt{3}} & 0 & 0\\ 0 & \frac{1}{\sqrt{6}} & 0\\ 0 & 0 & \frac{1}{\sqrt{2}} \end{array}\right]\left[\begin{array}{ccc} 1 & 1 & 1\\ 1 & 1 & -2\\ 1 & -1 & 0 \end{array}\right]\left[\begin{array}{c} L^{\prime }\\ M^{\prime }\\ S^{\prime } \end{array}\right]. \end{array}$$

We then calculate the mean and the standard deviation values of *l*, *α* and *β* for all target images and obtain the averaged mean and averaged standard deviation which are denoted as *μ*_*t*_^*l*^, *μ*_*t*_^*α*^, and *μ*_*t*_^*β*^, and *σ*_*t*_^*l*^, *σ*_*t*_^*α*^, and *σ*_*t*_^*β*^, respectively. These average mean and standard deviation values are calculated once and then used for the normalization of every input image. For each input image, we have to calculate the mean and standard deviation values denoted as *μ*_*s*_^*l*^, *μ*_*s*_^*α*^, and *μ*_*s*_^*β*^, and *σ*_*s*_^*l*^, *σ*_*s*_^*α*^, *σ*_*s*_^*β*^, respectively. 

The normalization of an input image is performed by calculating the new color values *l*′′, *α*′′ and *β*′′ for each pixel by the following equations:

(4) $$\begin{array}{c} l^{*}=l-\mu_{s}^{l},\;\;\alpha^{*}=\alpha -\mu_{s}^{\alpha },\;\;\beta^{*}=\beta -\mu_{s}^{\beta },\\ l^{\prime }=\frac{\sigma_{t}^{l}}{\sigma_{s}^{l}}l^{*},\;\;\alpha^{\prime }=\frac{\sigma_{t}^{\alpha }}{\sigma_{s}^{\alpha }}\alpha^{*},\;\;\beta^{\prime }=\frac{\sigma_{t}^{\beta }}{\sigma_{s}^{\beta }}\beta^{*},\\ l^{\prime \prime }=l^{\prime }+\mu_{t}^{l},\;\;\alpha^{\prime \prime }=\alpha^{\prime }+\mu_{t}^{\alpha },\;\;\beta^{\prime \prime }=\beta^{\prime }+\mu_{t}^{\beta }. \end{array}$$

Finally, we transform the resulting image in *lαβ* color space back to *RGB* color space by using (5):

(5) [RGB]=[4.4679−3.58730.1193−1.21862.3809−0.16240.0497−0.24391.2045] ×[exp⁡(33l′′+66α′′+22β′′)exp⁡(33l′′+66α′′−22β′′)exp⁡(33l′′−63α′′+0β′′)].

Figures 2(b) and 2(d) show the normalization results of Figures 2(a) and 2(c), respectively. The color distributions of the normalized images are comparable to those of the target images. All input images from different batches of specimens can be processed by this procedure for color normalization.

### 3.2. HSI Model Transformation and Three-Stepped Color Segmentation

**Figure 3:**
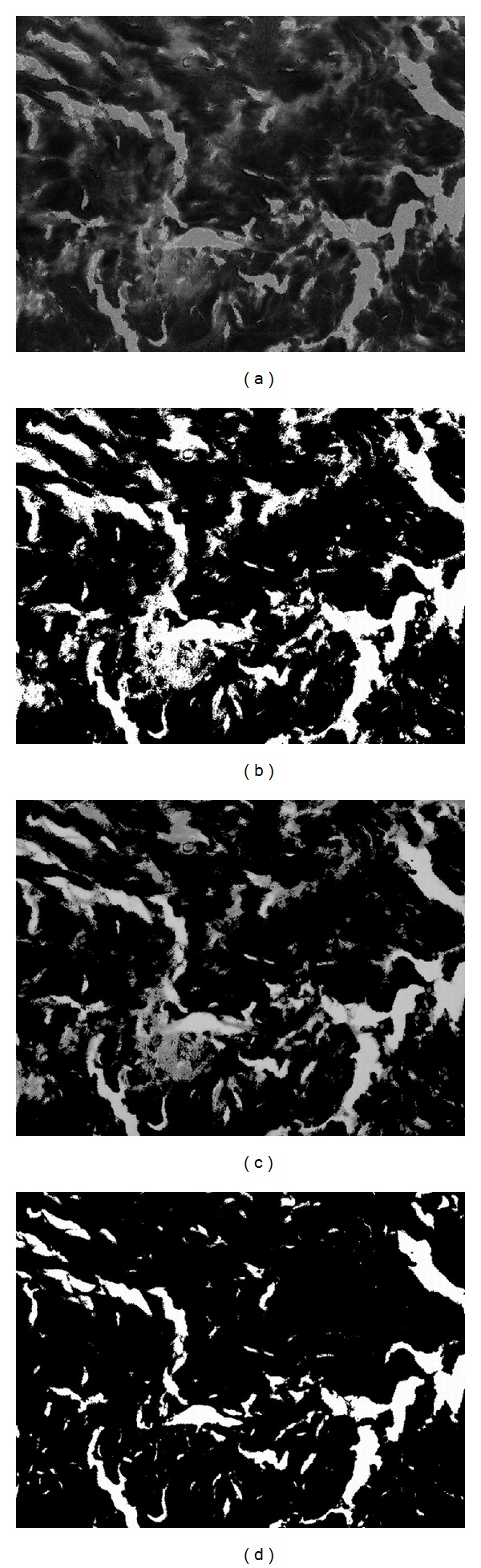
Color segmentation for Figure 2(a) (part 1). (a) The hue component of Figure 2(b); (b) the Otsu thresholding result of (a); (c) overlap the G channel to the white areas of (b); (d) the result of segmentation on (c), where white areas represent empty background and black areas represent tissue foreground.

Before color segmentation, we transform the normalized image into the *HSI* color space by using (6) [11, 12]. Currently, the pink part and major part of the purple areas in the normalized image are lower in hue value, and the background and some small parts of the purple areas in the normalized image have higher hue values.  Figure 3(a) shows the hue component of Figure 2(b):

(6) $$\begin{array}{c} H=\{\begin{array}{cc} \theta & \text{if}\;\;B\leq G\\ 360-\theta & \text{if}\;\;B>G, \end{array}\\ \theta =\cos^{-1}\left\{\frac{\left(1/2\right)\left[\left(R-G\right)+\left(R-B\right)\right]}{{\left[{\left(R-G\right)}^{2}+\left(R-B\right)\left(G-B\right)\right]}^{1/2}}\right\}. \end{array}$$

Based on the hue distribution, we apply the automatic thresholding method proposed by Otsu [13] to obtain the first binary image as shown in Figure 3(b), which is roughly divided into foreground and background. In Otsu's thresholding, the optimal threshold k*, which separates two classes, is obtained by using optimization:

(7) $$\begin{array}{c} \underset{k^{*}}{\arg\;\max}\left\{\sigma_{B}^{2}\left(k\right),0\leq k\leq 255\right\}, \end{array}$$

where *σ*_*B*_^2^ = *ω*_0_ *ω*_1_(*μ*_0_ − *μ*_1_)^2^ is the interclass variance and *w*_0_, *w*_1_, *μ*_0_, and *μ*_1_ are the probabilities of class occurrences and the mean levels of the two classes, respectively. The black areas represent the pink and most of the purple tissue areas as the foreground and the white areas cover some small parts of the purple tissue areas and the empty background. In other words, some purple areas may be faultily classified into the background. To make the foreground include all the purple tissues, we have to extract the remaining purple part from the background areas. The obtained background areas are used as the mask to map onto the G channel of normalized image, which is shown in Figure 3(c), and the second Otsu thresholding on the G channel is then applied to obtain the remaining purple areas. We then get the second binary image as in Figure 3(d), where the white area represents the real background and the black area represents the complete foreground of pink and purple tissue areas.

**Figure 4:**
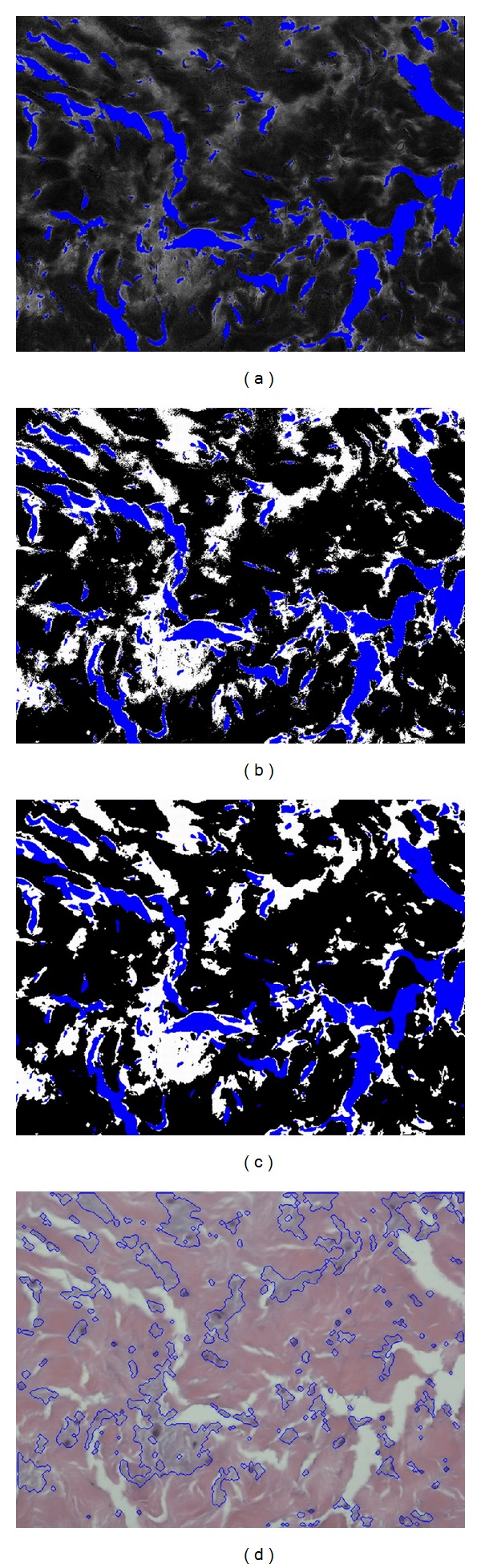
Color segmentation for Figure 2(a) (part 2). (a) Blue is the empty background and the other hue component areas are tissue foreground; (b) the segmented result, where blue represents background, white represents abnormal tissue, and black represents normal tissue; (c) rank filtering result; (d) boundaries of abnormal tissue regions mapped onto the normalized image.

After obtaining the foreground, we then have to separate the abnormal tissue from the normal tissue areas. In Figure 4(a), we label the background areas obtained in the previous step in blue and overlap onto the original hue component image in Figure 3(a). As mentioned before, the normal tissue areas show lower hue values and the abnormal areas have higher hue, so we can use the Otsu thresholding again to divide these two areas. The segmented result is shown in Figure 4(b), where the blue areas represent the background, the black areas represent the normal tissues, and the white areas represent the abnormal tissues. As the segmentation results are fragmented in the boundaries, we apply the rank filter to remove fragmented regions. We calculate the pixel numbers of each color in Figure 4(b) with a 9 × 9 mask and then assign the color with the highest count to the central pixel of mask; the result is shown in Figure 4(c).  Figure 4(d) shows the boundaries of abnormal tissues mapped onto the normalized image.

### 3.3. Active Double Thresholding and Nuclei Classification

Another characteristic to evaluate the level of pathological change is the ratio of round nuclei which belongs to the abnormal cells. We can use this ratio, instead of the area ratio, to characterize tissue condition when the staining colors are faded or if specimens are degraded after a long preservation time.

**Figure 5:**
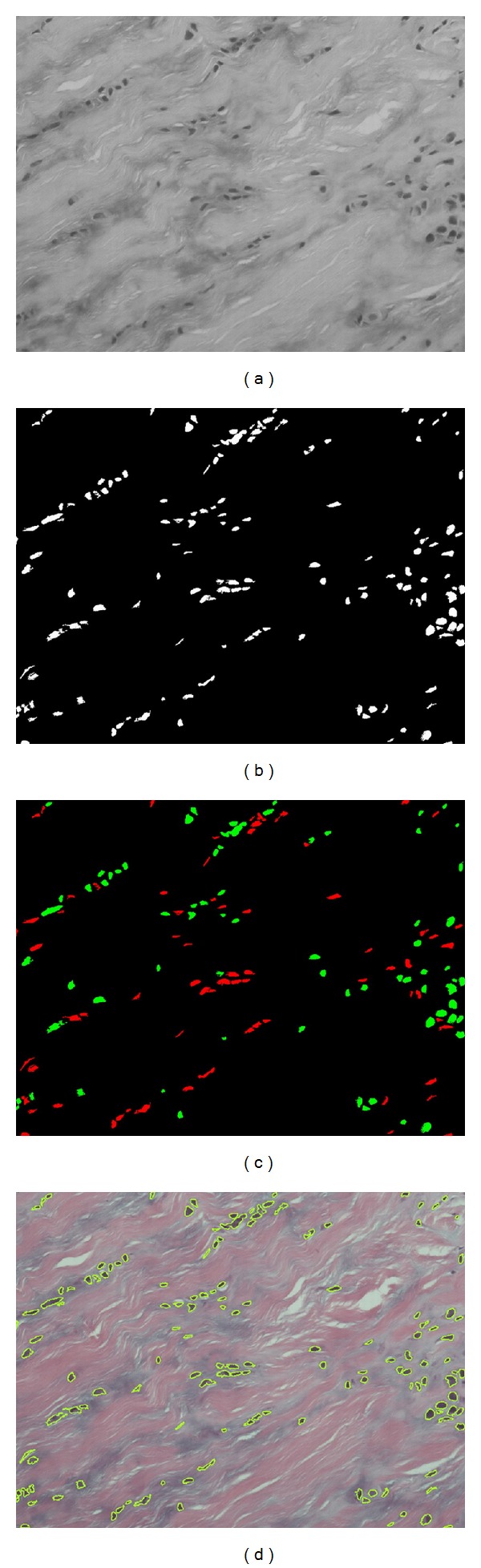
Nuclei classification for Figure 2(d). (a) The R channel of Figure 2(d); (b) the result after double thresholding; (c) the classification result, where red represents the normal nuclei and green represents the abnormal nuclei; (d) overlap the nucleus edges onto the original image.

After color normalization, we find that the R channel of the normalized image is more suitable for nuclei segmentation due to its high contrast of nuclei as in Figure 5(a). (In this section, we demonstrate the procedures of nuclei classification with another normalized image shown in Figure 2(d).) Therefore, we use the double thresholding scheme [14] to segment the nucleus areas. The intensity of nuclei is nearly the darkest of the whole R channel image. As the intensity distributions of images are different, we thus apply an active thresholding scheme to satisfy all images. First, for each input R channel image, we take the average of the ten lowest intensity values as the lowest intensity value of the image. Second, we add two empirical values 30 and 45 to this lowest value and use them as the two values for double thresholding. The lower threshold value is used as the seed and the higher threshold value is the restriction of region growing. After we apply the double thresholding scheme, the white areas of the resulting image represent the segmentation of nuclei and the segmentation result of nuclei is shown in Figure 5(b).

Now we can classify the segmented nuclei into three categories according to their shapes. The normal nuclei are usually long and rod-like, and the abnormal nuclei are usually round in shape. However, the connected area with multinuclei, which is regarded as the third category, is irregular in shape and classified as abnormal because only the abnormal nuclei will grow and connect each other into a cluster.

To classify these nuclei, we then calculate the area size, the circularity index, and the maximum and the minimum distances between the centroid and boundary points for each nucleus area. We then classify the nucleus as normal and rod-like if the circularity index is less than 0.95, the ratio of maximum to minimum distance is greater than 3, and the area is less than 2,000 pixels. All other areas are then classified as abnormal nuclei. In addition, we also define the area as a single abnormal round nucleus if the area is less than 2,000 pixels. 

After defining all the single abnormal round nuclei, we then calculate the average area of these nuclei. The average area is then used to calculate how many nuclei are in a connected multinuclei area.  Figure 5(c) shows the classification results where red presents the normal nuclei, green presents the abnormal nuclei. The nucleus edges were overlapped onto the original image as shown in Figure 5(d).

## 4. Results and Discussion

### 4.1. Specimen Preparation

In this study, we collected abnormal and normal specimens from trigger finger patients and nondiseased cadavers, respectively. All the specimens used in the experiments were graded into four severity stages as H (High), M (Middle), L (Low), and N (Normal) in trigger finger disease by the pathologist (Dr. Hsiao-Bai Yang). The numbers of collected specimens were 10 with H stage, 10 with M stage, 6 with L stage, and 3 with N stage, respectively (29 specimens in total). From each specimen, 49 images in the size of 2560 × 1920 were acquired by using our previously developed autofocusing system [15]. As some of the 49 images contained a large area of background and irrelevant tissues (e.g., microvasculature), such images provided less image evidence of pulley tissues and were not suitable for evaluating the proposed pathological parameters. Consequently, the same pathologist of our research group was asked to exclude the unsuitable images based on her expertise on tissue pathology. Then, a random selection process was performed to acquire 10 images from the remaining images for the subsequent quantitative analysis. 

### 4.2. Pathological Indices

The proposed microscopic image analysis system was designed to obtain two pathological parameters. The size ratio of abnormal tissue regions is parameter 1 which can be calculated by using (8). In (8), the area of normal tissue regions represents the sum of pink (or blue for abnormal) areas from the 10 selected images of each specimen.  Table 1 presents the resulting parameter 1 s for different specimens obtained by using the proposed color segmentation procedure. The number ratio of abnormal nuclei is parameter 2 and can be calculated by using (9). In (9), the number of normal (or abnormal) nuclei is the total number of normal (or abnormal) nuclei obtained from the 10 selected images of each specimen by using the rule-based classifier.  Table 2 shows the resulting parameter 2 s for different specimens:

(8) The  size  ratio  of  abnormal  tissue  regions  =Area  of  abnormal  tissue  regionsArea  of  abnormal  tissue  regions+Area  of  normal  tissue  regions

(9) The  number  ratio  of  abnormal  nuclei  =Number  of  abnormal  nucleiNumber  of  abnormal  nuclei+Number  of  normal  nuclei.

**Figure 6:**
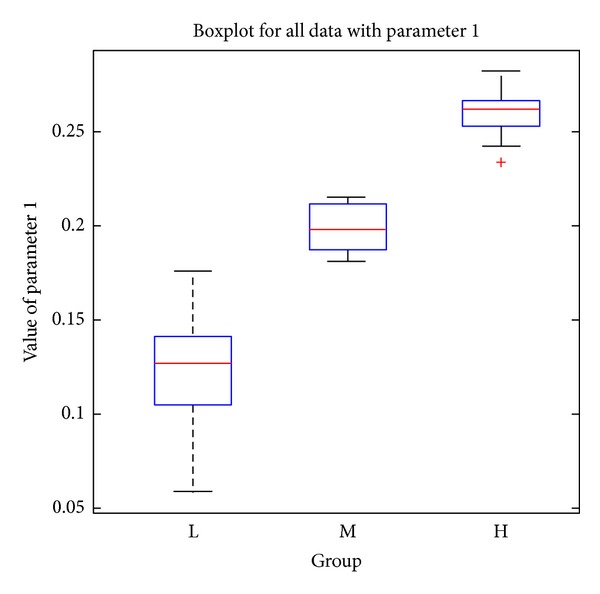
Boxplot for the three severity stages with parameter 1.

**Figure 7:**
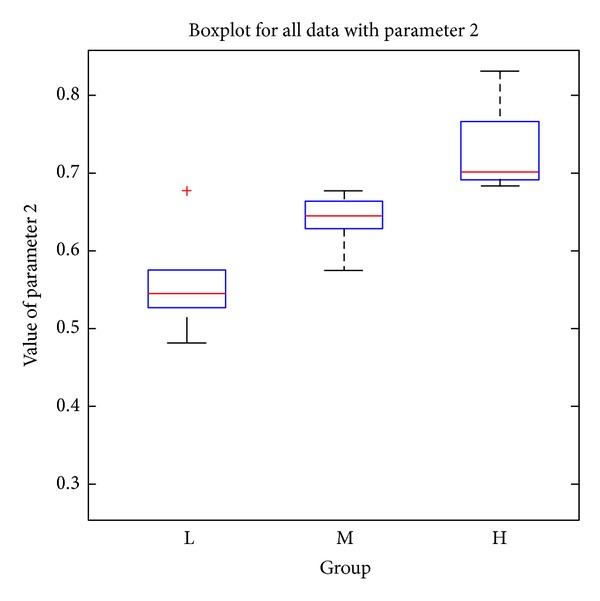
Boxplot for the three severity stages with parameter 2.

Based on the pathological staging, the resulting parameters in Table 1 show clear deviations among the three (H, M, and L) stages. There are significant differences between the mean values of adjacent stages. Using the average of two mean values for two adjacent stages, we can obtain two threshold values to perform simple discrimination between the three severity stages. Consequently, there are no errors in H and M stages and only one misclassification from L to M stage from all the collected specimens of our experiments. In Table 2, the number ratio of abnormal nuclei also shows similar characteristics with good deviations among the three stages. Simple discrimination among the three severity stages is performed the same way as in Table 1. There are no classification errors in the H stage, 2 misclassifications from M to L stage, and 1 misclassification from L to M stage. However, the three misclassifications with parameter 2 have no intersections with the one with parameter 1. This implies that the discrimination between three severity stages can be correctly performed with the weighted combination of parameters 1 and 2. Since we only have a limited number of specimens presently, a more complicated classification mechanism is left for research with more sufficient specimens in the future. The pathological parameters of N stage are measured with only 3 specimens and also presented in Tables 1 and 2. The mean values of the two parameters are all smaller than the ones of L stage. Because stages L and N are less severe, the resulting measurements reflect the clinical expectation. Figures 6 and 7 show the boxplots [16] for the three severity stages with parameter 1 and parameter 2, respectively. For each box in the figure, the central mark is the median, the edges of box are the 25th and 75th percentiles, the whiskers extend to the most extreme data points, and outliers are plotted individually. The boxplots also reflect the clustering ability of the two parameters similar to the above-mentioned simple discrimination examples.

In addition, statistical analysis was performed by Student's *t*-test and the *P* values between different severity stages were calculated (as shown in Table 3). If the *P* value is less than 0.05, the two groups are considered to have significant differences and can be easily divided. For parameter 1, the *P* values for group pairs H versus M and M versus L are 0.000 and 0.004, respectively. For parameter 2, the *P* values for group pairs H versus M and M versus L are 0.000 and 0.028, respectively. As all statistical tests are significant (less than 0.05), it is suggested that both parameters can be used as pathological indices for grading the severity stages effectively.

### 4.3. System Performance

#### 4.3.1. Parameter Setting

The values of the system parameters used in active double thresholding (in Section 3.3) could be a factor influencing the stability of automated image analysis. Thus, we employed the color normalization step to effectively reduce the influences caused by different imaging and staining conditions. After color normalization, the system parameters can be determined based on the intensity contrast between pulley tissue and surrounding regions on the normalized images. When applying the same parameter values throughout the entire experiment with 290 images, the proposed system was capable of achieving accurate measurement results.

On the other hand, the system parameters used in nuclei classification were determined and tuned by the pathologists based on their pathological knowledge and clinical experiences. Our experimental results showed that the system is capable of making correct discriminations between the disease stages based on the ratio of abnormal nuclei by using the same set of system parameters. All the 290 images from the 29 specimens were analyzed consistently. If some more complicated parameters are designed for tissue measurement in the future, more complex classifiers can be helpful to determine these system parameters.

#### 4.3.2. Computational Time

The system was developed on an Intel Core i5 2.8 GHz PC with 3.5 GB memory. For an image of 2560 × 1920 pixels, the average computational time of color normalization, color segmentation, and nuclei classification was approximately 5, 12, and 10 seconds, respectively. 

## 5. Conclusions

In this paper, we have developed an automatic image analysis system to evaluate the severity of trigger finger disease from the microscopic pulley images. Two pathological parameters are designed and can be computed automatically and efficiently. The quantitative measurements are stable and without intra- and interoperator variability of manual measurements. Twenty-nine pulley specimens are evaluated with the same image analysis setting in the experiments. The experimental results show that the two parameter measures have good deviations among the three pathological stages and can be used to discriminate the severity stages with simple discrimination mechanism. Thus, the proposed image analysis system clearly provides an efficient and reliable way in measuring the pathological progression of trigger finger disease. The quantitative parameters are objective and can also be extended for other kinds of pathological specimens. In the future, we will recruit more cases in the validation of trigger finger disease and also explore new opportunities for other clinic applications.

## Figures and Tables

**Table 1:** The size ratio of abnormal tissue regions (parameter 1).

Specimen no.	Normal (pixel^2^)	Abnormal (pixel^2^)	Ratio
H-1	31791895	12495997	0.282
H-2	30894213	10986876	0.262
H-3	31869804	11038359	0.257
H-4	32511973	11026116	0.253
H-5	33797170	10267132	0.233
H-6	31635397	11474053	0.266
H-7	31451368	11224479	0.263
H-8	32950410	11695269	0.262
H-9	34067450	10887277	0.242
H-10	27747278	10290969	0.271

Mean ± SD			0.259 ± 0.014

M-1	35698059	7946874	0.182
M-2	34326147	8515580	0.199
M-3	35324719	8461582	0.193
M-4	33284256	7671304	0.187
M-5	34422738	9418395	0.215
M-6	34315916	9170559	0.211
M-7	33027627	9072248	0.215
M-8	34051300	7529957	0.181
M-9	35167293	8706583	0.198
M-10	32745438	8083811	0.198

Mean ± SD			0.198 ± 0.013

L-1	40491940	5972857	0.129
L-2	43877544	2773360	0.059
L-3	36923582	6077911	0.141
L-4	37539086	4417329	0.105
L-5	34975123	7460192	0.176
L-6	36623101	5244448	0.125

Mean ± SD			0.123 ± 0.039

N-1	40904631	5482052	0.118
N-2	32792539	3323970	0.092
N-3	32724101	4340358	0.117

Mean ± SD			0.109 ± 0.054

**Table 2:** The number ratio of abnormal nuclei (parameter 2).

Specimen no.	Normal	Abnormal	Ratio
H-1	271	660	0.709
H-2	481	1088	0.693
H-3	244	529	0.684
H-4	117	385	0.767
H-5	289	1098	0.792
H-6	721	1653	0.696
H-7	292	655	0.692
H-8	165	817	0.832
H-9	446	1013	0.694
H-10	59	151	0.719

Mean ± SD			0.728 ± 0.051

M-1	382	687	0.643
M-2	318	591	0.650
M-3	192	360	0.652
M-4	131	187	0.588
M-5	308	652	0.679
M-6	173	343	0.665
M-7	335	456	0.576
M-8	726	1311	0.644
M-9	382	647	0.629
M-10	379	795	0.677

Mean ± SD			0.640 ± 0.034

L-1	824	771	0.483
L-2	1080	1210	0.528
L-3	520	636	0.550
L-4	404	477	0.541
L-5	862	1165	0.575
L-6	123	259	0.678

Mean ± SD			0.559 ± 0.066

N-1	473	247	0.343
N-2	310	205	0.398
N-3	292	308	0.513

Mean ± SD			0.418 ± 0.074

**Table 3:** *P* values between different serious stages.

Group pair	Parameter 1	Parameter 2
High versus middle	0.000	0.000
Middle versus low	0.004	0.028
